# Esters of valerenic acid as potential prodrugs

**DOI:** 10.1016/j.ejphar.2014.03.019

**Published:** 2014-07-15

**Authors:** Juliane Hintersteiner, Maximilian Haider, Denise Luger, Christoph Schwarzer, Gottfried Reznicek, Walter Jäger, Sophia Khom, Marko D. Mihovilovic, Steffen Hering

**Affiliations:** a Department of Pharmacology and Toxicology, University of Vienna, Althanstrasse 14, A-1090 Vienna, Austria; b Institute of Applied Synthetic Chemistry, Vienna University of Technology, Getreidemarkt 9, A-1060 Vienna, Austria; c Department of Pharmacology, Innsbruck Medical University, Peter-Mayr-Straße 1, 1a A-6020 Innsbruck, Austria; d Department of Pharmacognosy, University of Vienna, Althanstrasse 14, A-1090 Vienna, Austria; e Department of Clinical Pharmacy and Diagnostics, University of Vienna, Althanstrasse 14, A-1090 Vienna, Austria

**Keywords:** GABA_A_ receptors, Valerenic acid derivatives, Behavioral analysis, 2-Microelectrode-voltage-clamp-technique, LC–MS/MS

## Abstract

Valerenic acid (VA) is a β_2/3_ subunit-specific modulator of γ-aminobutyric acid (GABA) type A (GABA_A_) receptors inducing anxiolysis. Here we analyze if VA-esters can serve as prodrugs and if different ester structures have different in vitro/in vivo effects. Modulation of GABA_A_ receptors expressed in *Xenopus* oocytes was studied with 2-microelectrode-voltage-clamp. Anxiolytic effects of the VA-esters were studied on male C57BL/6N mice by means of the elevated plus maze-test; anticonvulsant properties were deduced from changes in seizure threshold upon pentylenetetrazole infusion. VA was detected in plasma confirming hydrolysis of the esters and release of VA in vivo. Esterification significantly reduced the positive allosteric modulation of GABA_A_ (α_1_β_3_γ_2S_) receptors in vitro. in vivo, the studied VA-ester derivatives induced similar or even stronger anxiolytic and anticonvulsant action than VA. While methylation and propylation of VA resulted in faster onset of anxiolysis, the action of VA-ethylester was longer lasting, but occurred with a significant delay. The later finding is in line with the longer lasting anticonvulsant effects of this compound. The estimated VA plasma concentrations provided first insight into the release kinetics from different VA-esters. This might be an important step for its future clinical application as a potential non-sedative anxiolytic and anticonvulsant.

## 1 Introduction

γ-Aminobutyric acid (GABA) type A receptors (GABA_A_) are the major inhibitory neurotransmitter receptors in the mammalian brain. GABA_A_ receptors belong to the superfamily of Cys-loop-type ligand-gated ion channels (Olsen and Sieghart, 2008). Nineteen GABA_A_ receptor subunits have been identified in the human genome, comprising α_1–6_, β_1–3_, γ_1–3_, δ, ε, θ, π and ρ_1–3_ (Simon et al., 2004). Five receptor subunits form a chloride-selective ion channel. Receptor activation opens the channel and induces transmembrane chloride currents (*I*_GABA_) modulating neuronal excitability and transmitter release (Sieghart, 2006; Sigel and Steinmann, 2012). There is consensus that the major adult receptor isoform consists of 2α_1_, 2β_2_ and one γ_2_ subunit (Olsen and Sieghart, 2008).

GABA_A_ receptors play a major role in the treatment of central nervous system (CNS) diseases such as generalized anxiety and panic disorders, epilepsy, and sleep disturbances (Möhler, 2006). They are the molecular target of the classical benzodiazepines (e.g. diazepam) and subtype-selective benzodiazepine site ligands such as zolpidem or zopiclone, barbiturates, anaesthetics, and anticonvulsants (Sigel and Steinmann, 2012). Beside these drugs, GABA_A_ receptors are modulated by multiple natural products (Johnston et al., 2006).

We and others have shown that valerenic acid (VA), a constituent of *Valeriana officinalis*, enhances *I*_GABA_ through GABA_A_ receptors. VA binds with nanomolar affinity (Benke et al., 2009) and modulates GABA_A_ receptors in an allosteric manner. VA selectively interacts with receptors comprising β_2/3_-subunits (Benke et al., 2009; Khom et al., 2007). A point mutation in the β_2_-subunit (N265S) of recombinant receptors prevents *I*_GABA_ enhancement while the “reversed mutation” in β_1_ (S266N) enhances current stimulation to extents observed on β_2/3_-subunit containing receptors (Khom et al., 2007). Valerenic acid induces anxiolysis in the elevated plus maze and the light/dark choice test in mice (Benke et al., 2009; Khom et al., 2010). Anxiolysis was absent in β_3_(N265M) point-mutated mice supporting the hypothesis that the anxiolytic effects of VA are caused by interaction with β_3_-containing GABA_A_ receptors (Benke et al., 2009).

A recently published pharmacokinetic study on rats revealed that approximately 34% of VA are absorbed after oral administration with a half-life between 2.7 and 5 h (Sampath et al., 2012). This good bioavailability is in line with the reported anxiolysis of VA after oral administration in mice (Benke et al., 2009).

Together these findings make VA or one of its derivatives (Khom et al., 2010; Kopp et al., 2010) interesting drug candidates. Little is known, however, how this molecule penetrates the blood-brain barrier (Neuhaus et al., 2008). Ester prodrugs can enhance the lipophilicity (by masking charged groups such as carboxylic acids) and thereby affect the time course of drug action (Beaumont et al., 2003). Therefore four VA-esters (VA-methylester (VA-ME), VA-ethylester (VA-EE), VA-propylester (VA-PE) and VA-pivaloyloxymethylester (VA-POM)) have been synthesized in order to address the following questions about the biological activity of these potential prodrugs: (i) Does esterification affect modulation of *I*_GABA_ through GABA_A_ receptors? (ii) Do VA-esters represent prodrugs (i.e. are esters hydrolyzed in vivo and is VA detectable in the plasma? (iii) Are VA-esters active in vivo and – if so – does esterification affect the anxiolytic and anticonvulsant properties of VA?

## 2 Materials and methods

All experiments on animals were carried out in accordance to the Austrian Animal Experimental Law, which is in line with the EU directive 2010/63/EU.

### 2.1 Chemicals

**Fig. 1:**
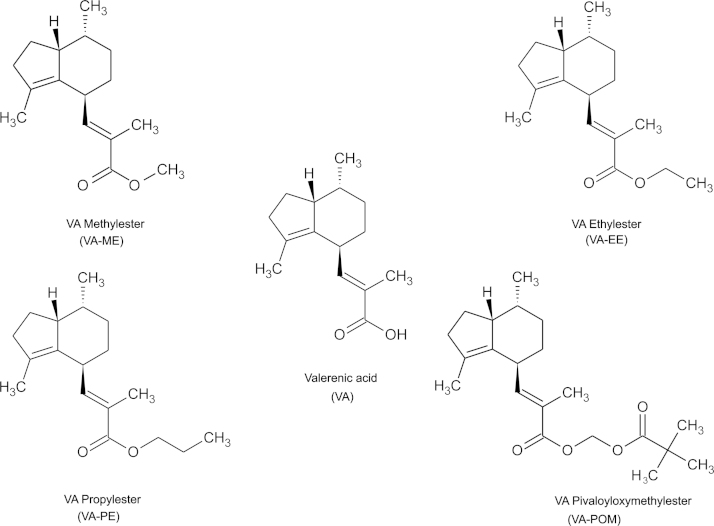
Structural formulae of VA and synthesized ester derivatives.

Valerenic acid (VA) was purchased from HWI Pharma Solutions (Rülzheim, Germany) and converted into the aforementioned derivatives as described below (for structural formulae see Fig. 1). Chemicals used in this study were obtained from Sigma-Aldrich (Vienna, Austria) except where otherwise stated. Dichloromethane (DCM), dimethylsulfoxide (DMSO), formic acid, methanol and *t*-butylmethylether were of p.a. quality and purchased from ROTH (Karlsruhe, Germany). For HPLC analysis double distilled water and acetonitrile, HPLC quality (VWR Int., Vienna, Austria) were used.

LogP values for the aimed compounds were calculated using ACD/ChemSketch freeware.

All reactions were carried out in oven dried 4 ml-reaction vials under an argon atmosphere. DCM was predistilled and then desiccated on Al_2_O_3_ columns (PURESOLV, Innovative Technology; Amesbury, USA). Reaction mixtures were magnetically stirred and monitored by thin layer chromatography using Merck Silica 60F_254_ plates (Merck, Vienna, Austria). Flash chromatography was performed on a Sepacore Flash System (2× Büchi Pump Module C-605, Büchi Pump Manager C-615, Büchi UV Photometer C-635, Büchi Fraction Collector C-660; Büchi Labortechnik, Flawil, Switzerland) using Merck silica gel (0.040–0.063 mm, 230–400 mesh). Yields refer to chromatographically and spectroscopically pure compounds. ^1^H-NMR (200 MHz) and ^13^C-NMR (50 MHz) were recorded on Bruker AC 200 (200 MHz; Bruker, Karlsruhe, Germany). The chemical shifts δ are reported relative to the residual solvent peaks. All ^1^H and ^13^C shifts are given in ppm (s=singulet, d=doublet, t=triplet, q=quadruplet, m=multiplet). Specific rotation was measured on an Anton Paar MCP500 polarimeter (Anton Paar GmbH; Graz, Austria) at 20 °C in DCM.

LC–MS/MS analyses were carried out on an Ultimate 3000 RSLC-series system (Thermo Fisher Scientific Austria, Vienna, Austria) coupled to a triple quadrupol mass spectrometer API 4000 (AB Sciex Instruments, Framingham, USA).

### 2.2 Synthesis of valerenic acid esters

#### 2.2.1 Valerenic acid methylester (VA-ME)

Valerenic acid (30.0 mg, 1 Eq., 0.13 mmol) and 4-dimethylaminopyridine (DMAP, 1.6 mg, 0.1 Eq, 0.01 mmol) were dissolved in 1.3 ml dry DCM under an Argon atmosphere and cooled to 0 °C, then 1-ethyl-3-(3-dimethylaminopropyl)carbodiimide (EDCI; 36.8 mg, 1.5 Eq, 0.19 mmol) was added in one portion. After stirring the mixture for five min methanol (23.7 μl, 4.5 Eq, 0.59 mmol) was added dropwise and the mixture was left warming to room temperature overnight. The solution was taken up in 50 ml ethylacetate (EtOAc) and was subsequently washed with saturated NH_4_Cl solution (three times), saturated NaHCO_3_ solution (three times) and once with brine; it was then dried and concentrated under reduced pressure. Purification of the crude material via column chromatography (LP:EtOAc=30:1) provided 30.2 mg (94%) of Valerenic acid methylester as a colorless oil.

• ^**1**^**H-NMR (200 MHz, CDCl**_**3**_**):** *δ*=0.77 (d, *J*_1_=7.0, 3*H*), 1.37–1.98 (m, 14*H*), 2.19 (t, *J*_1_=7.5 Hz, 1*H*), 2.90–2.99 (m, 1*H*), 3.49–3.56 (m, 1*H*), 3.72 (s, 3*H*), 7.01 (dq, *J*_1_=1.4 Hz, *J*_2_=9.8 Hz, 1*H*)
• ^**13**^**C-NMR (50 MHz, CDCl**_**3**_**)** *δ*=12.0 (s), 12.4 (s), 13.5 (s), 24.5 (d), 25.4 (d), 28.7(d), 33.0 (t), 34.3 (t), 37.4 (d), 47.4 (t), 51.7 (s), 125.7 (q), 130.9 (q), 133.4 (t), 169.0 (q)

Analytical data is consistent with the reported data for Valerenic acid methylester (Kopp et al., 2010).

#### 2.2.2 Valerenic acid ethylester (VA-EE)

Using the analogous procedure as for the preparation of VA-ME, treatment of valerenic acid (20.0 mg, 1 Eq, 0.09 mmol) with 4-dimethylaminopyridine (1.0 mg, 0.1 Eq, 0.009 mmol), EDCI (24.4 mg, 1.5 Eq, 0.13 mmol) and ethanol (22.3 μl, 4.5 Eq, 0.38 mmol) yielded 20.2 mg (95%) of VA-EE as colorless oil.

• ^**1**^**H-NMR (200 MHz, CDCl**_**3**_**, ppm):** *δ*=0.78 (d, *J*=7.0, 3*H*), 1.29 (t, *J*=7.1 Hz, 3*H*), 1.37–2.02 (m, 14*H*), 2.19 (t, *J*=7.6 Hz, 2*H*), 2.92–2.98 (m, 1*H*), 3.46–3.56 (m, 1*H*), 4.17 (q, *J*=7.1 Hz, 2*H*), 7.01 (dq, *J*_1_=9.8 Hz, *J*_2_=1.4 Hz, 1*H*)
• ^**13**^**C-NMR (50 MHz, CDCl**_**3**_**, ppm):** *δ*=12.0 (s), 12.4 (s), 13.5 (s), 14.3 (s), 24.5 (d), 25.5 (d), 28.7(d), 33.1 (t), 34.3 (t), 37.4 (d), 47.4 (t), 60.4 (d), 125.7 (q), 130.9 (q), 133.4 (t), 169.0 (q)

Analytical data is consistent with the reported data for Valerenic acid ethylester (Kopp et al., 2010).

#### 2.2.3 Valerenic acid propylester (VA-PE)

Using the analogous procedure as for the preparation of VA-ME, treatment of valerenic acid (30.0 mg, 1 Eq, 0.13 mmol) with 4-DMAP (1.6 mg, 0.1 Eq, 0.01 mmol), EDCI (36.8 mg, 1.5 Eq, 0.19 mmol) and propanol (28.5 μl, 4.5 Eq, 0.38 mmol) yielded 35.1 mg (99%) of VA-PE as colorless oil.

• ^**1**^**H-NMR (200 MHz, CDCl**_**3**_**, ppm):** *δ*=0.77 (d, *J*=7.0, 3*H*), 0.95 (t, *J*=7.4 Hz, 3*H*), 1.37–2.01 (m, 16*H*), 2.19 (t, *J*=7.7 Hz, 2*H*), 2.91–2.97 (m, 1*H*), 3.48–3.55 (m, 1*H*), 4.07 (t, *J*=6.7 Hz, 2*H*), 7.02 (dq, *J*_1_=9.8 Hz, *J*_2_=1.3 Hz, 1*H*)
• ^**13**^**C-NMR (50 MHz, CDCl**_**3**_**, ppm)** *δ*=10.5 (s), 12.0 (s), 12.4 (s), 13.5 (s), 24.5 (d), 25.5 (d), 28.7(d), 33.1 (t), 34.3 (t), 37.4 (d), 47.4 (t), 66.0 (d), 126.0 (q), 130.7 (q), 133.5 (q), 143.3 (d) 168.7 (q)

[α]^20^_D_=−77.0 (*c*=0.2, DCM);

#### 2.2.4 Valerenic acid pivaloyloxymethylester (VA-POM)

Valerenic acid (20 mg, 1 Eq, 0.13 mmol) and 1,8-diazabicycloundec-7-ene (21.4 μL, 1.1 Eq, 0.14 mmol) were dissolved in dry DCM (1.3 ml) under an argon atmosphere; pivaloyloxymethyl chloride (20.2 μl, 1.1 Eq, 0.14 mmol) was added dropwise (Urban et al., 2005). The mixture was stirred overnight until full conversion before it was taken up in 50 ml EtOAc and subsequently washed three times with saturated NH_4_Cl and NaHCO_3_ solution and one time with brine. The organic phase was dried and concentrated in vacuo. Purification by column chromatography (LP:EtOAc=4:1) furnished 19.1 mg (85%) of VA-POM as a slightly yellow oil.

• ^**1**^**H-NMR (200 MHz, CDCl**_**3**_**, ppm):** *δ*=0.78 (d, *J*=6.9, 3*H*), 1.21 (s, 9*H*), 1.38–1.98 (m, 15*H*), 2.20 (t, *J*=7.4 Hz, 2*H*), 2.91–2.95 (m, 1*H*), 3.50–3.55 (m, 1*H*), 5.81 (s, 2*H*), 7.09 (dq, *J*_1_=9.8 Hz, *J*_2_=1.1 Hz, 1*H*)
• ^**13**^**C-NMR (50 MHz, CDCl**_**3**_**, ppm)** *δ*=12.0 (s), 12.2 (s), 13.5 (s), 24.5 (d), 25.3 (d), 26.8 (s), 28.7(d), 33.0 (t), 34.3 (t), 37.4 (d), 38.8 (q), 47.4 (t), 79.9 (d), 125.0 (q), 131.2 (q), 133.1 (q), 145.6 (d) 167.0 (q), 177.2(q)

[α]^20^_D_=−70.4 (*c*=0.6, DCM);

### 2.3 Expression and functional characterization of GABA_A_ receptors

Preparation of stage V–VI oocytes from *Xenopus laevis* (Nasco, Ft. Atkinson, WI), synthesis of capped off run-off poly(A^+^) cRNA transcripts from linearized cDNA templates (pCMV vector) was performed as described (Khom et al., 2006). Briefly, female *Xenopus laevis* were anaesthetized by exposing them for 15 min to a 0.2% solution of MS-222 (methane sulfonate salt of 3-aminobenzoic acid ethyl ester), before surgically removing parts of the ovaries. Follicle membranes from isolated oocytes were enzymatically digested with 2 mg/ml collagenase (Type 1 A). One day after isolation, the oocytes were injected with about 10–50 nl of diethylpyrocarbonate—treated water containing the different cRNAs at a concentration of approximately 150–3000 ng/μl/subunit. The amount of cRNA was determined by means of a NanoDrop ND-1000 (Kisker-biotech, Steinfurt, Germany). To ensure expression of the γ-subunit in the case of α_1_β_3_γ_2S_ receptors, cRNAs were mixed in a ratio of 1:1:10 (Boileau et al., 2002). Oocytes were stored at 18 °C in ND96 solution (Methfessel et al., 1986). Electrophysiological experiments were conducted using the two-microelectrode voltage-clamp method at a holding potential of −70 mV using a TURBO TEC 01C amplifier (npi electronic, Tamm, Germany) and an Axon Digidata 1322 A interface (Molecular Devices, Sunnyvale, CA) applying pCLAMP v. 9.2 data acquisition. The bath solution consisted of 90 mM NaCl, 1 mM KCl, 1 mM MgCl_2_·6H_2_O, 1 mM CaCl_2_ and 5 mM 2-(4-(2-hydroxyethyl)-1-piperazinyl)-ethanesulfonic acid (HEPES; pH 7.4). Microelectrodes were filled with 2 M KCl and had resistances between 1 and 3 M Ω (Khom et al., 2006).

### 2.4 Perfusion system

GABA and the tested compounds were applied by means of fast perfusion system (Baburin et al., 2006). Drug or control solutions were applied by means of a TECAN Miniprep 60 (npi electronic, Tamm, Germany) permitting automation of the experiments. To elicit *I*_GABA_ the chamber was perfused with 120 µl of GABA-containing solution at volume rate between 300 and 1000 µl/s. The *I*_GABA_ rise time ranged between 100 and 250 ms. To account for possible slow recovery from increasing levels of desensitization in the presence of high compound concentrations, the duration of washout periods was extended from 1.5 min (GABA EC_3–7_) to 3 min (co-application of GABA EC_3–7_ and 1 µM compound) to 5–10 min (co-application of GABA EC_3–7_ and 10 µM compound) to 15–20 min (co-application of GABA EC_3–7_ and 30 µM compound) to 30 min (GABA EC_3–7_ and 100 µM compound). Oocytes with maximal current amplitudes >3 µA were discarded to exclude voltage-clamp errors (Khom et al., 2006).

### 2.5 Analyzing concentration–response curves

Stimulation of chloride currents by modulators of the GABA_A_ receptor was measured at a GABA concentration eliciting between 3 and 7% of the maximal current amplitude (EC_3–7_). The EC_3–7_ was determined at the beginning of each experiment.

Enhancement of the chloride current was defined as (*I*_(GABA+Compound)_/*I*_GABA_)−1, where *I*_(GABA+Compound)_ is the current response in the presence of the compound and *I*_GABA_ is the control GABA current. Each data point represents the mean±S.E.M from at least 5 oocytes and ≥2 oocyte batches.

### 2.6 Behavioral analysis

#### 2.6.1 Animals

Male mice (C57BL/6N) were obtained from Charles River Laboratories (Sulzfeld, Germany). For maintenance, mice were group-housed (maximum 5 mice per type IIL cage) with free access to food and water. At least 24 h before the commencement of experiments, mice were transferred to the testing facility, where they were given free access to food and water. The temperature in the maintenance and testing facilities was 22±2 °C; the humidity was 40–60%; a 12 h light-dark cycle was in operation (lights on from 07.00 to 19.00). Only male mice – aged 3–6 months – were tested. Compounds at a dose of 3 mg/kg bodyweight or solvent alone were applied by intraperitoneal (i.p.) injection. The dose was chosen according to a previously published dose–response curve (Khom et al., 2010). Testing solutions were prepared in a solvent composed of saline (0.9% NaCl solution with 10% DMSO and 3% Polysorbat 80). The final DMSO concentration was fixed to 10% (Broadwell et al., 1982). Application of the solvent alone did not influence animal behavior.

#### 2.6.2 Elevated plus maze (EPM) test

The animals’ behavior was tested over 5 min on an elevated plus maze 1 m above ground consisting of two closed and two open arms, each 50×5 cm in size. The test instrument was built from grey PVC; the height of closed arm walls was 20 cm. Illumination intensity was set to 180 lx. Animals were placed in the center, facing an open arm. Analysis of open and closed arm entries, distance and time on open arm was automatically done with Video-Mot 2 equipment and software (TSE systems, Bad Homburg, Germany). Drugs or solvent were applied 15, 30 or 60 min before testing.

#### 2.6.3 Seizure threshold

Seizure threshold was determined by pentylenetetrazole (PTZ) tail-vein infusion on freely moving animals at a rate of 100 µl/min (10 mg/ml PTZ in saline). Infusion was stopped when animals displayed generalized clonic seizures. Animals were killed by cervical displacement immediately after the first generalized seizure. The seizure threshold dose was calculated from the infused volume in relation to body weight. The compounds were injected 15, 30, 60, 90, 120 or 150 min before PTZ infusion. At the infusion rate of 100 µl/min, generalized seizures are induced within 90 s.

### 2.7 Detection of free VA in the plasma

#### 2.7.1 Sampling

Blood samples were taken 15, 30, 60 and 120 min after i.p. injection of the compounds. 10 min after i.p injection of thiopental (150 mg/kg bodyweight in 0.9% sodium chloride solution) blood samples (500–800 µl) were collected and compiled into ethylenediamine tetra-acetic acid (EDTA)-coated micro tubes (1.6 mg EDTA/sample) and centrifuged at 12,000 rpm for 5 min at 4 °C. Plasma samples were transferred into 1.5 ml tubes and stored at −80 °C until analysis.

#### 2.7.2 Sample preparation

A liquid–liquid extraction method together with an internal standard (IS) acetoxyvalerenic acid (ACVA; PhytoLab GmbH&Co KG, Vestenbergsgreuth, Germany) was applied for the quantification of VA in plasma. To 100 µl of plasma sample 10 µl of IS solution (1 µg/ml 10% aqueous DMSO) was added. These solutions were extracted by liquid–liquid partition with 400 µl of dichloromethane/*t*-butylmethylether (80:20, v/v) and vortexed for 5 min. From the clear lower organic layer the solvent was removed through a constant nitrogen stream at room temperature (25 °C). The residue was dissolved with 100 µl of methanol, sonicated, centrifuged for 5 min (15,000 rpm) and the supernatant was finally transferred to autosampler vials (Macherey-Nagel vial N9, 0.2 ml with integrated insert; Macherey-Nagel, Düren, Germany).

#### 2.7.3 Quantification of valerenic acid by LC–MS/MS

The samples (10 µl) were analyzed by liquid chromatography/mass spectrometry (LC–MS/MS) on an Ultimate 3000 RSLC-series system (Thermo Fisher Scientific Austria, Vienna, Austria) coupled to a triple quadrupol mass spectrometer (AB Sciex Instruments API 4000) equipped with an orthogonal APCI source operated in negative mode and displayed with Analyst 1.5 software.

LC separation was performed on an Acclaim RSLC 120C18 column (3 µm, 150×2.1 mm I.D., Thermo Fisher Scientific Austria, Vienna, Austria), preceded by an Acclaim 120C18 guard cartridge (5 µm, 10×2 mm I.D., Thermo Fisher Scientific Austria, Vienna, Austria), at a flow rate of 0.500 ml/min and a column temperature of 30 °C. The mobile phase consisted of a continuous linear gradient, mixed from aqueous formic acid, pH 3.5 (mobile phase A), and acetonitril (mobile phase B), to elute VA. The gradient ranged from 50% B (0 min) to 80% at 8 min, kept constant at 80% until 10 min, and finally decreased linearly to 50% again at 11 min. Between sampling, the column was purged with 98% B (acetonitrile) for 4 min before equilibrating for 6 min resulting in a total analysis time of 18 min. Within this setup valerenic acid eluted at 4.06 min., acetoxyvalerenic acid (IS) at 7.02 min. Selective and sensitive detection and quantification was carried out using MS/MS fragmentation of VA resp. acetoxyvalerenic acid (ACVA) giving a quasimolecular ion at *m*/*z* 233 [M–H]^−^ (VA) and *m*/*z* 291 [M−H]^−^ (ACVA). MRM *m*/*z* 233/84 (VA) as well as *m*/*z* 291/249 (ACVA) were used for calibration curves to give a linear concentration range from 0.1 ng/ml (LLOD, *S*/*N*=4) to 500 ng/ml (correlation coefficient 0.9996). Extraction efficiencies (average 84%) were determined by comparison of peak areas between quality control (QC) and analysis samples. For validation, quality control (QC) samples were prepared in the same way as the calibration standards.

The triple quadrupol mass spectrometer operated with the following parameters: APCI neg., NC-5, CUR 10, GS1 30, GS2 18, TEM 400 °C, CAD 12, EP-11, DP-65, CXP-5, CEM 2100, DF 200. MRM *m*/*z* 233/84 (VA): CE-29, dwell time 300 ms. MRM *m*/*z* 291/249 (ACVA): CE-24, dwell time 300 ms.

### 2.8 Statistical analysis

Statistical significance of electrophysiological data was calculated using a paired Student *t*-test; for in vivo experiments, one-way ANOVA (followed by posthoc Bonferroni analysis) was used. Statistical analysis was done with Origin software (OriginLab Corporation; USA). *P*-values of <0.05 were accepted as statistically significant. All data are given as mean±S.E.M.

## 3 Results

### 3.1 *I*_GABA_ modulation by VA-esters

**Fig. 2:**
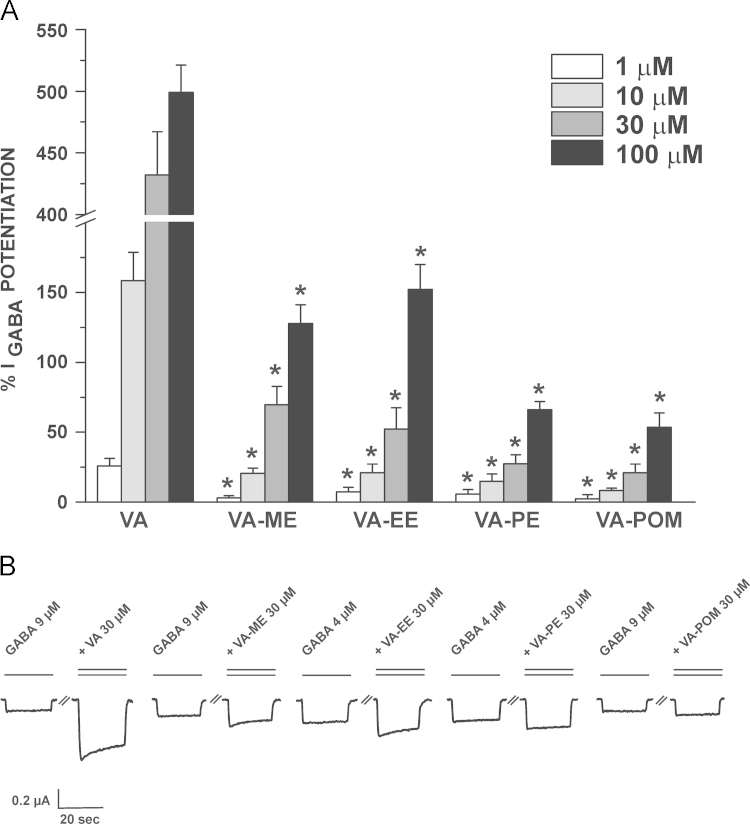
*I*_GABA_ modulation by VA-ester derivatives **(A)** Enhancement of *I*_GABA_ through GABA_A_ receptors composed of α_1_β_3_γ_2S_ subunits by 1 µM (white bars), 10 µM (light grey bars), 30 μM (dark grey bars) and 100 μM (black bars) of the indicated compounds. Each value represents the mean±S.E.M from at least 5 oocytes and ≥2 oocyte batches. (^*^) indicates significantly different from *I*_GABA_ enhancement by VA at the same concentration (*P*<0.05, Student׳s *t*-test) **(B)** Typical traces for the potentiation of chloride currents through α_1_β_3_γ_2S_ channels by VA-derivatives at a GABA EC_3–7_. Control currents (GABA, single bar) and corresponding currents elicited by co-application of GABA and the indicated compound (double bar) are shown.

Fig. 1 displays the structures of the studied VA-derivatives (see Section 2 for synthesis). As expected, *I*_GABA_ modulation by VA-esters was less pronounced than by VA. This is shown in Fig. 2 illustrating modulation of *I*_GABA_ through α_1_β_3_γ_2S_ GABA_A_ receptors during co-application of GABA (EC_3–7_) and either VA, VA-ME, VA-EE, VA-PE or VA-POM.

VA-ME and VA-EE induced significantly stronger *I*_GABA_ enhancement than VA-PE and VA-POM (max. *I*_GABA_ potentiation _(VA-ME 30 µM)_: 70±13% (*n*=5) and max. *I*_GABA_ potentiation _(VA-EE 30 µM)_: 52±15% (*n*=6) vs. max. *I*_GABA_ potentiation _(VA-PE 30 µM)_: 27±6% (*n*=5) and max. *I*_GABA_ potentiation _(VA-POM 30 µM)_: 21±6% (*n*=6). Compared to VA the modulation of *I*_GABA_ at 30 µM was drastically reduced (from 6.2-fold (VA-ME) to 20.6-fold reduction (VA-POM)). At 1 µM none of the studied ester derivatives induced significant *I*_GABA_ enhancement (Fig. 2 A).

### 3.2 Anxiolytic action of VA-esters

For investigation of the time course of in vivo activity of the different derivatives, effects on anxiety-related behavior were tested 15, 30 and 60 min after i.p. application of either solvent (=control) or drug containing solutions at a dose of 3 mg/kg bodyweight.

**Fig. 3:**
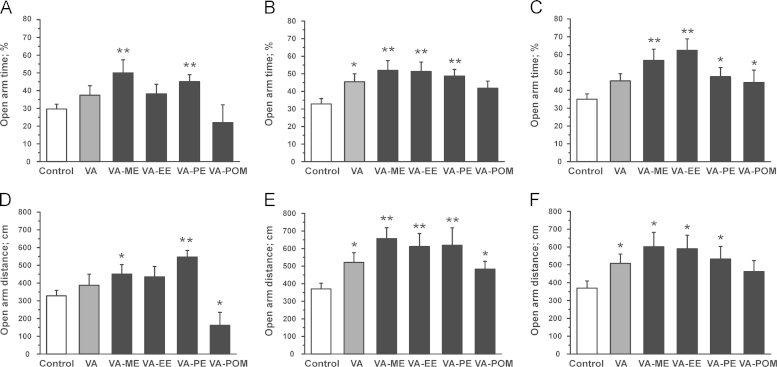
Effects on explorative behavior of VA-esters in the elevated plus maze test are compared to saline-treated control (white bars) mice at a dose of 3 mg/kg bodyweight. Bars display the time spent (in % of the total time) on the open arms (**(A)**–**(C)**) and the open arm distance (**(D)**–**(F)**) 15 (left column), 30 (mid column) and 60 (right column) min after i.p. application of the indicated compounds. Each bar represents a mean±S.E.M from at least 8 different mice. (^⁎^) indicates statistically significant differences with *P*<0.05, (^⁎⁎^) with *P*<0.01 to control.

**Fig. 4:**
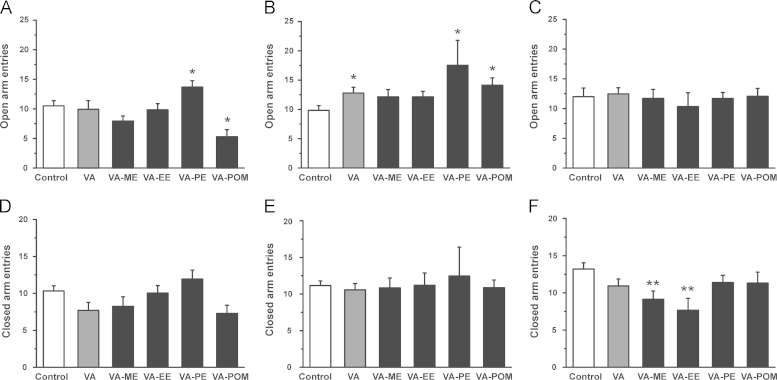
Number of entries to the open (OA; **(A)**–**(C)**) and closed arms (CA; **(D)**–**(F)**) of the elevated plus maze 15 (left column), 30 (mid column) and 60 (right column) min after i.p. application of the indicated compound are compared to control (white bars) at a dose of 3 mg/kg bodyweight. Each bar represents a mean±S.E.M from at least 8 different mice. (^⁎^) indicates statistically significant differences with *P*<0.05, (^⁎⁎^) with *P*<0.01 to control.

As illustrated in Fig. 3A, 15 min after injection, control mice spent 29.7±2.7% of the total time (*n*=33) in the open arms (OA) of the EPM. An increase of time spent in the OA was observed upon application of VA-ME and VA-PE (VA-ME: 50.0±7.3%; *n*=16, *P*<0.01; VA-PE: 45.1±3.9%; *n*=19; *P*<0.01) Animals treated with VA-esters also covered significantly longer distances on the OA (Control: 327.8±30.8 cm; *n*=33 vs. VA-ME: 451.1±53.4 cm; *n*=16, *P*<0.05 vs. VA-PE: 546.7±37.1 cm; *n*=19, *P*<0.01, see Fig. 3D) suggesting anxiolytic activity. No significant effects on time spent in the OA, covered distance on the OA, OA and CA entries were observed for VA-EE (Figs. 3 and 4A and D). Animals treated with VA-POM covered a significantly shorter distance on the OA (Fig. 3D) and displayed fewer OA entries (Fig. 4A) compared to control littermates, while no significant effect on time spent on the OA and CA entries was observed (Fig. 3A and Fig. 4D).

30 min after injection mice treated with VA, VA-ME, VA-PE and VA-EE spent significantly more time in the OA compared to control mice (control: 32.8±3.1%; *n*=19 vs. VA: 45.5±4.6%; *n*=14; *P*<0.05 vs. VA-ME: 51.9±5.5%; *n*=13; *P*<0.01; VA-EE: 51.3±5.4%; *n*=15; *P*<0.01; VA-PE: 48.7±3.7%; *n*=17; *P*<0.01; see Fig. 3B). These increase in time spent in the OA was accompanied by longer distances covered on the OA compared to control animals (Control: 370.4±32.4 cm; *n*=19 vs. VA: 521.2±55.1 cm; *n*=14, *P*<0.05 vs. VA-ME: 655.9±62.6 cm; *n*=13, *P*<0.01; VA-EE: 611.8±73.7 cm; *n*=15, *P*<0.01; VA-PE: 618.3±100.0 cm; *n*=17, *P*<0.05; Fig. 3E). Mice treated with VA and VA-PE also visited the OA more frequently, while no effect on OA visits upon VA-ME and VA-EE application was observed (Fig. 4B). Interestingly, significantly increased ambulation on the OA (482.6±44.9 cm; *n*=15, *P*<0.05; Fig. 3E) and a higher number of OA visits (Fig. 4B) were also observed for mice treated with VA-POM compared to control littermates. However, time spent on the OA did apparently not differ significantly from control (Fig. 3B). No differences in the number of CA entries between control and compound treated mice were observed 30 min after application (Fig. 4E).

As illustrated in Figs. 3 and 4C, 60 min after injection, the exploratory drive (time spent in OA and OA entries) in mice treated with VA was not significantly different from control animals, although mice covered a longer distance on the OA (Control: 369.6±40.3 cm; *n*=15 vs. VA: 508.2±52.5 cm; *n*=17, *P*<0.05; Fig. 3F). In contrast, application of VA-ME, VA-EE and VA-PE induced increased ambulation of open arms also 60 min after treatment (VA-ME: 56.7±6.3%; *n*=14; *P*<0.01; VA-EE: 62.9±7.2%; *n*=8; *P*<0.05; and VA-PE: 47.6±5.1%; *n*=10; *P*<0.05; Fig. 3C) accompanied by longer distances on the OA (VA-ME: 601.0±81 cm.2; *n*=14, *P*<0.05 vs. VA-EE: 589.8±76.1 cm; *n*=8, *P*<0.05 vs. VA-PE: 532.7±70.5 cm; *n*=10, *P*<0.05; Fig. 3F). Furthermore, while the number of OA entries did not differ from control, the number of CA entries significantly dropped upon treatment with VA-ME and VA-EE compared to control mice (Fig. 4C and F). Weaker, yet significant effects on time spent in the OA were also observed for mice treated with VA-POM (44.4±7.0%; *n*=12; *P*<0.05), while the other parameters did significantly not differ from control (Figs. 3 and 4C and F).

No significant changes in total distance were observed for any drug at any time point, suggesting no sedative effects at this dose.

### 3.3 Anticonvulsant action of VA-esters

Loreclezole, a GABA_A_ receptor modulator selective for β_2/3_ subunits, displays in vivo anticonvulsant activity (Greenfield, 2013; Groves et al., 2006; Sanna et al., 1996; Wingrove et al., 1994). It was therefore interesting to study if VA and the ester derivatives would induce comparable effects.

**Fig. 5:**
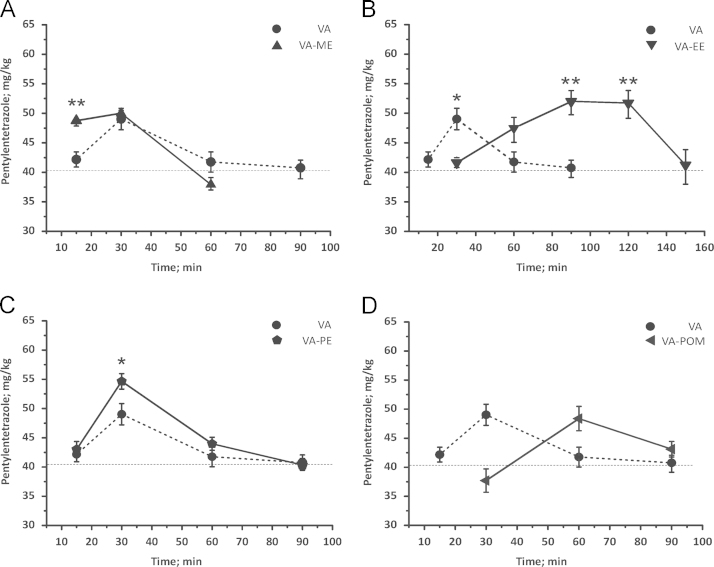
Changes in seizure threshold upon PTZ-infusion are compared at a dose of 3 mg/kg bodyweight of VA (dotted line) and **(A)** VA-ME, **(B)** VA-EE, **(C)** VA-PE and **(D)** VA-POM. Each data point represents the mean±S.E.M from at least 3 mice; (^⁎^) indicates statistically significant differences with *P*<0.05; (^⁎⁎^) indicates statistically significant differences with *P*<0.01 to VA.

Application of VA induced an increased threshold against pentylenetetrazole (PTZ)-induced seizures 30 min after application of VA (control: 39.5±2.8 mg/kg; *n*=7 vs. VA: 49.0±1.8 mg/kg; *n*=4; *P*<0.05). No anticonvulsant effect was observed either at 15 or 60 min after VA application (see Fig. 5A). In contrast to VA, VA-ME significantly increased seizure threshold already 15 min after application (48.8±0.5 mg/kg; *n*=4; *P*<0.01). The anticonvulsant effect persisted 30 min after treatment (50.0±0.5 mg/kg; *n*=3; *P*<0.05), however, VA-ME did not induce any significant effects on seizure threshold 60 min after application.

In contrast to VA, VA-EE did not induce anticonvulsant effects until 60 min after drug treatment (control: 39.5±2.8 mg/kg; *n*=7 vs. 47.5±2.4 mg/kg; *n*=3; *P*<0.05). Seizure threshold was further significantly increased 90 min after application (52.0±2.3 mg/kg; *n*=4; *P*<0.05) and remained at the same level even 120 min after application (51.7±2.6 mg/kg; *n*=4; *P*<0.05). 150 min after application the seizure threshold of VA-EE-treated mice did not differ from the control (see Fig. 5B).

As illustrated in Fig. 5C, VA-PE’s anticonvulsant activity was comparable to VA: Seizure threshold was significantly elevated 30 min after compound application (control: 39.5±2.8 mg/kg; *n*=7 vs. VA-PE: 54.7±1.3 mg/kg; *n*=4; *P*<0.05). The seizure threshold elevation at this time point was even more pronounced than that of VA or the other derivatives (*P*<0.05). However, no statistically significant anticonvulsant effects could be detected at a later time point.

No significant changes in seizure threshold were observed upon application of VA-POM until 60 min. At this time point VA-POM significantly increased seizure threshold (control: 39.5±2.8 mg/kg; *n*=7 vs. VA-POM: 48.4±2.1 mg/kg; *n*=4; *P*<0.05). No anticonvulsant activity, however, was observed 90 min after application (Fig. 5D).

As shown in Table 1 high concentrations of free VA could be detected in plasma samples already after 15 min indicating rapid hydrolysis of the VA-esters.

## 4 Discussion

Valerenic acid, from *V. officinalis*, is an efficient modulator of GABA_A_ receptors. VA binds with nanomolar affinity, modulates *I*_GABA_ at low micromolar concentrations and causes anxiolysis in rodents with little sedation (Benke et al., 2009; Khom et al., 2010, 2007). VA specifically interacts with receptors containing β_2_ and β_3_ subunits (Benke et al., 2009; Khom et al., 2007). A point mutation in the β_3_ subunit (N265M) prevents anxiolytic activity of VA in mice. It was therefore concluded that anxiolysis occurs via neurons expressing β_3_ comprising GABA_A_ receptors (Benke et al., 2009). Subunit-selective ligands like VA would be expected to exhibit a selective therapeutic profile with fewer side effects and may thus represent an interesting lead structure for the development of novel GABA_A_ receptor modulators (Atack, 2011a, 2011b, 2010; Möhler, 2012).

Little is known, however, if different VA-esters with higher lipophilicity would have different anxiolytic and anticonvulsant action. Neuhaus et al. (2008) hypothesized that VA does not permeate the blood-brain barrier by passive diffusion through the lipid bilayer but rather *via* a paracellular transport route.

Therefore, we have now performed a proof-of-concept study to test if masking the carboxylic acid of VA by esterification (Fig. 1) would affect the in vivo activity of VA. Four VA-esters with different lipophilicity (LogP: VA (5.13±0.31)<VA-ME (5.64±0.28)<VA-EE (6.17±0.28)<VA-PE (6.70±0.28)<VA-POM (6.97±0.40), Fig. 1; calculated using ACD/ChemSketch freeware) were designed and their anxiolytic and anticonvulsant activity subsequently analyzed.

As expected, esterification of VA significantly reduced *I*_GABA_ modulation (Fig. 2), which was evident for all 4 tested derivatives. None of the VA-esters increased *I*_GABA_ at 1 µM, while significant stimulation was induced by VA (Fig. 2).

It is expected that the esters are transformed into the highly active VA by esterases ubiquitously found in the blood, liver, brain and other organs and tissues (Liederer and Borchardt, 2006). This assumption is in line with the observed in vivo action of the VA-esters (VA-ME, VA-EE and VA-PE, Figs. 3–5) and was directly confirmed by estimation of the plasma concentrations of VA (Table 1).

In order to obtain information about potential differences in the time courses of anxiolytic activity of esters, behavior was analyzed 15, 30, 60 min after treatment. As shown in Figs. 3 and 4, methylation and propylation of VA resulted in a faster onset of anxiolysis, while VA-EE and VA-POM displayed no activity after 15 min. All esters were almost equally active after 30 min with the exception of the VA-POM that did not cause significant anxiolysis (Fig. 3B). A longer lasting anxiolytic action of VA-ME and VA-EE is evident from Fig. 3C where both compounds were at 60 min significantly more active than VA. These alterations may depend on different factors including distinct distribution of the prodrug into organs dependent on its lipophilicity. This may also include alterations of binding to plasma proteins or fatty tissue. A second important factor is the conversion of the non-active prodrug into the active VA by esterases.

The anticonvulsant activity of VA and the ester derivatives was observed for the first time. Significant differences in onset and length of the anticonvulsant action of the VA-esters are evident from Fig. 5. VA (dotted line in Fig. 5A–D) displayed little activity after 15 min, reached maximal anticonvulsant activity after 30 min that decayed until 90 min. A comparable transient time course of action was observed for the propylester (VA-PE) displaying, however, significantly stronger effects at 30 min. An exceptional result was obtained with the ethylester of VA: remarkably, VA-EE displayed no significant anticonvulsant activity at early time points (15 and 30 min, Fig. 5B). However, a long-lasting anticonvulsant action of this compound until 120 min is evident from Fig. 5B. The anticonvulsant action of VA-POM was similarly delayed but not so long lasting as VA-EE (compare Fig. 5B and D). The late anticonvulsant effects of VA-EE (Fig. 5B) are in line with its pronounced anxiolytic effect after 60 min (Fig. 3C). In contrast, VA-ME displayed highest anxiolytic and anticonvulsant activities at the earliest time-point (15 min). The most lipophilic VA-POM displayed the least anxiolytic or anticonvulsant activity compared to the most polar VA. This might relate to two possible explanations: either the ethylester is stronger bond to proteins or lipophilic surfaces, leading to a slower but longer distribution, or the ethylester is less accessible for esterases, leading to an increased stability of this potential prodrug. The first reason appears unlikely, because the lipophilicity of the ethylester is comparable to those of the methyl- and propylesters, which both display faster onset of effects. Of note is the fact, that the most lipophilic ester (VA-POM) displayed comparatively little in vivo activity, suggesting that this compound might be trapped in lipophilic structures.

Hydrolysis of VA-esters in plasma was confirmed employing an LC–MS/MS assay as already described (Sampath et al., 2012). Plasma levels after i.p. application of any of the VA-esters after 15 min were lower than after application of VA despite the stronger in vivo activity (Figs. 3–5). Tissue binding and delayed hydrolysis of VA-esters by esterases might contribute to the lower plasma levels of VA.

However, in vivo VA-esters were similarly or even more active than VA (VA-ME, VA-EE, VA-PE) (see Figs. 3–5) which may indicate a rapid penetration of these potential prodrugs into brain. Based on their lipophilicity it cannot be excluded that parent VA-esters may reach significant brain concentration. Although esterification of VA strongly reduces *I*_GABA_ modulation in vitro (Fig. 2) their potentially better penetration of the blood–brain barrier may contribute to the overall anxiolytic and anticonvulsant activity. The much slower onset and longer lasting anticonvulsant activity of VA-EE and VA-POM may reflect a slower hydrolysis in the brain (see Fig. 5B and D). Ongoing animal studies shall therefore verify time- and dose-dependent penetration of VA-esters into brain.

Taken together, several VA-esters display similar or stronger in vivo activity than VA. The different time courses of anticonvulsant activity (e.g. fast onset of the VA-ME and long lasting effects of the VA-EE) may be beneficial for potential therapeutic use of this molecule. Future studies will show whether the increased lipophilicity of the esters will affect the oral bioavailability of VA.

## Figures and Tables

**Table 1:** Plasma concentrations of free VA; 15, 30 and 60 min after application of VA and VA-esters (3 mg/kg bodyweight) are indicated in ng/ml. Each data point represents the mean±S.E.M of 4 animals per group.

**Compound**	**15 min**	**30 min**	**60 min**
VA	640.7±131.8	105.2±18.6	61.3±21.8
VA-ME	164.2±47.2	76.7±19.7	24.2±4.3
VA-EE	117.4±19.3	84.1±11.3	20.2±4.1
VA-PE	274.5±51.8	174.6±52.3	43.7±6.7
VA-POM	166.5±9.1	80.4±27.1	11±1.5
